# Raman microspectroscopy as a biomarking tool for in vitro diagnosis of cancer: a feasibility study

**DOI:** 10.3325/cmj.2012.53.551

**Published:** 2012-12

**Authors:** Aleksandra Pavićević, Sofija Glumac, Jelena Sopta, Ana Popović-Bijelić, Miloš Mojović, Goran Bačić

**Affiliations:** 1 University of Belgrade, Faculty of Physical Chemistry, Belgrade, Serbia; 2 University of Belgrade, Faculty of Medicine, Institute of Pathology, Belgrade, Serbia

## Abstract

**Aim:**

To elucidate whether Raman spectroscopy aided by extensive spectral database and neural network analysis can be a fast and confident biomarking tool for the diagnosis of various types of cancer.

**Methods:**

Study included 27 patients with 11 different malignant tumors. Using Raman microscopy (RM) a total of 540 Raman spectra were recorded from histology specimens of both tumors and surrounding healthy tissues. Spectra were analyzed using the principal component analysis (PCA) and results, along with histopathology data, were used to train the neural network (NN) learning algorithm. Independent sets of spectra were used to test the accuracy of PCA/NN tissue classification.

**Results:**

The confident tumor identification for the purpose of medical diagnosis has to be performed by taking into account the whole spectral shape, and not only particular spectral bands. The use of PCA/NN analysis showed overall sensitivity of 96% with 4% false negative tumor classification. The specificity of distinguishing tumor types was 80%. These results are comparable to previously published data where tumors of only one tissue type were examined and can be regarded satisfactorily for a relatively small database of Raman spectra used here.

**Conclusion:**

In vitro RM combined with PCA/NN is an almost fully automated method for histopathology at the level of macromolecules. Supported by an extensive tumor spectra database, it could become a customary histological analysis tool for fast and reliable diagnosis of different types of cancer in clinical settings.

Raman spectroscopy (RS) and infrared spectroscopy (IR) have been used extensively in studying biological molecules. The potential of these techniques to become complementary to standard histology has recently intensified the application of RS and IR in the analysis of biological tissues (1,2). The advantage of these techniques over classical histology is that they do not require staining of samples (histology without chemicals) and that the acquisition of spectra can be implemented almost automatically and interpreted by computer-based algorithms. RS is based on inelastic interaction between light and matter by which the molecule’s vibrational state is raised (3). When the molecule returns to its background level, a photon is emitted at a different wavelength from the incident light (Raman shift). All Raman shifts provide a Raman spectrum that is directly related to the molecular composition of the tissue creating a molecular fingerprint whereas the intensity of the Raman peaks is directly proportional to the concentration of specific molecules.

Promising results have been reported for in vitro, ex vivo, and in vivo assessments of various human tumors in a variety of organs such as the skin, cervix, lungs, breasts, bladder, brain, liver, kidneys, nasopharynx, etc (1,4-11). RS studies are frequently performed as a comparison of spectra of healthy and affected tissues combined with histological analysis, which is then used to classify measured spectra as tumors or non-tumors and/or to distinguish between different tumors. However, there are inherent problems involved in the analysis of spectra:

(i) Raman scattering from tissues is inherently weak and frequently overlapped by the undesirable endogenous fluorescence since the cross-section for a typical tissue fluorophore is an order of a million times larger than that of Raman scattering. Fluorescence removal techniques are widely exploited by using different background subtraction algorithms (11,12), but this method is not always preferable since potentially significant background information could be overlooked (6). As a potential solution, Raman microspectroscopy (RM) has been recently introduced (13). This is a technique that uses a specially designed Raman spectrometer with an integrated optical microscope enabling the inspection of a sample and acquirement of Raman spectra of selected microscopic areas of larger samples thus avoiding areas of unwanted fluorescence.

(ii) biological tissues are rich in various biomolecules (lipids, proteins, nucleic acids) each having its corresponding set of Raman peaks whose spectral band assignments have been extensively reviewed and presented as the wide list of over 1000 chemical shifts (1). Although Raman peaks are spectrally narrow, a vast number of spectral features that are frequently contained in an overall Raman signal of biological tissues results in signal overlapping creating broad signal envelopes. Hence, it is almost impossible to typify different tissues by the traditional procedure of finding some peaks that are specific for certain tissue, especially when attempting to differentiate cancerous from neighboring normal tissue. Consequently, a comprehensive mathematical analysis of the whole spectrum has to be used, instead of analyzing individual peaks one-by-one and assigning certain peaks as a tissue fingerprint. One of the most widespread mathematical techniques is the principal component analysis (PCA) of raw spectra, which has shown to be useful for data analysis of tumor samples by grouping Raman peaks (8,9,14,15). The other method commonly used is the application of the neural network (NN) algorithm for the Raman signal post-processing (16-19). Using this approach, a number of Raman spectra of various histologically defined tissue samples was used for NN training in terms of learning the spectral patterns. The performance of the NN is then evaluated on an independent set of spectra, by prediction of lesion type and comparing it to the true class. It is also possible to use PCA data for feeding NN (16,17).

In this study we applied both advanced developments of modern Raman spectroscopy described above. We acquired Raman spectra from 11 different tumor types along with neighboring healthy tissue using RM to avoid areas with unwanted high fluorescence. The combined PCA and NN mathematical approach was applied to analyze and classify spectral data obtained by RM measurements. The majority of tumors were soft and bone tumors, the types that have not yet been investigated by RS. The more important aim of this study was to investigate the feasibility of creating a RS tissue database containing tumors of different origin which can be used for Raman spectroscopy based histology under circumstances that are likely to occur in clinical settings (eg, biopsies or *ex tempora* analysis).

## Methods

### Tissue samples

The study included 27 patients with 11 different pathohistological diagnoses. All samples were obtained from the Faculty of Medicine, Institute of Pathology of the University of Belgrade. Fresh, non-fixed biological material was selected for frozen section analysis (fixation creates artifacts in RS) (20). Tissue samples were routinely cut in the cryostat at -20°C in 50-μm thick slices for routine histopathology analysis. All slides were stored at -20°C for 1 hour before RM recordings were performed. Tissue samples included in this study were lung tumors (squamous carcinoma, adenocarcinoma, and small cell carcinoma), bone and soft tissue tumors (chondrosarcoma, chondroblastoma, osteosarcoma, Ewing's sarcoma, rhabdomyosarcoma, and synovial sarcoma), malignant peripheral nerve sheath tumor (MPNST), and hepatocellular carcinoma, all paired with surrounding healthy tissues on separate slides.

### Experimental procedure

The Raman spectroscopy measurements were performed using a DXR Raman Microscope (Thermo Scientific Instruments Group, Waltham, MA USA), applying the following parameters: exposition time 20-second, number of acquisitions 10, laser wavelength 532 nm, laser power 10 mW, aperture 50 µm, magnification 50, average spot size 2.1 μm. For spectra acquisition and background fluorescence correction, the software package OMNIC (Thermo Scientific) was used. Each tissue sample was recorded more than 10 times by focusing the laser at separate spots using a microscope. Dark spots in a sample were avoided since they usually gave unwanted fluorescence (probably from blood vessel areas containing hemosiderin). Signals obtained from these locations are generally useless since the signal from the background fluorescence is dominant and is of an order of a million times larger than that of Raman scattering. Even pre-irradiation (or bleaching) of samples was not helpful for obtaining acceptable spectra (data not shown). Finally, 10 spectra of each tissue sample were selected for further analysis. Altogether 540 spectra (270 for tumors and 270 for healthy tissues) were collected.

### Data analysis

The mathematical method for analysis and classification of raw spectra was applied using the Raman processing (RP) program for the MATLAB environment, developed at the SSIM/CARES Research Laboratory at Wayne State University (21). In the first step, PCA was executed and in the second NN training was performed resulting in the creation of a tumor spectra database.

*PCA.* The idea of this method is to decompose the whole spectra into factors, or principal components (PC), which represent the most common variations of the original data (22). Each PC is related to the spectrum with a variable called the score, representing the weight of that exclusive component in the basis spectrum. All 540 spectra were used for PCA and as a result the dimension of spectra was reduced to 8 most significant principal components.

*NN analysis*. A neural network is a computer program resembling a chain of neural cells, which could be trained to recognize even small changes in spectra not matching the standard spectrum (23). Neural networks are applied for solving problems where the relationships are complex or unknown. Half of all PCA data (randomly selected), linked with results of histological analysis were used as a NN training set, while the other half was used for testing the performance of the NN trained database. It has to be noted that the evaluation of successful classification of tumors, ie, tumors vs normal tissue (sensitivity) and differentiation among tumor types (specificity) was performed against the complete database including all tissues and all tumor types.

## Results

**Figure 1:**
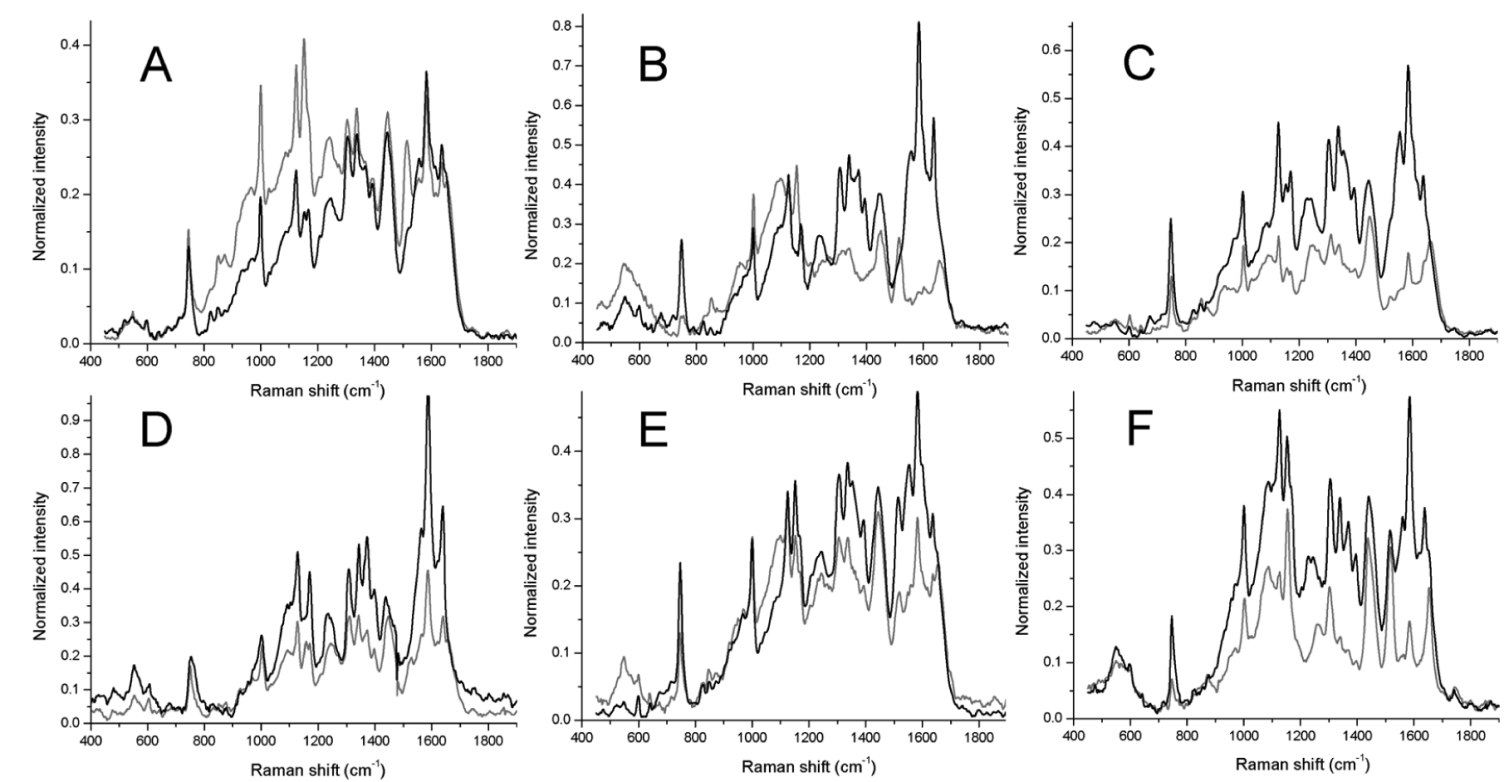
Examples of the mean Raman spectra of different types of diseased (gray line) and surrounding healthy (black line) tissues: (**A**) chondrosarcoma, (**B**) malignant peripheral nerve sheath tumor, (**C**) lung squamous carcinoma, (**D**) lung adenocarcinoma, (**E**) rhabdomyosarcoma, and (**F**) hepatocellular carcinoma.

The mean Raman spectra of selected tissue samples (normal and tumor) were obtained by simple averaging of 10 spectra (Figure 1). Individual spectral shapes of particular tissue, obtained by using microscope guided area selection, had essentially the same spectral bands, but with the slightly different signal intensities of some spectral bands. Nevertheless, even visual inspection of mean spectra showed differences in spectral shapes and band intensities between tumors and corresponding healthy tissues. The same was true for different tumors within the same tissue (compare tumor spectra on C and D); all of these indicating structural alterations of macromolecules.

**Figure 2:**
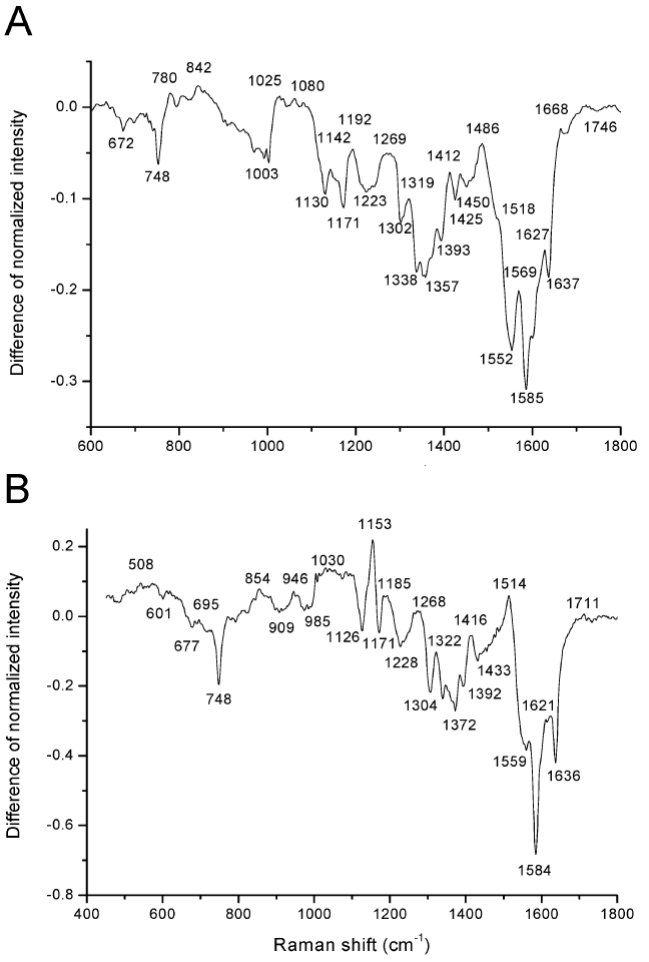
Raman spectral bands obtained by the subtraction of normalized spectra of tumor and surrounding healthy tissues for: (**A**) lung adenocarcinoma and (**B**) malignant peripheral nerve sheath tumor. Wavenumbers were obtained by the automatic find peak procedure.

Obtained spectra clearly showed that Raman shifts of both tumor and healthy tissues were densely compacted, making the tissue specific assignation of peaks a difficult process using the traditional interpretation of the spectra by peak assignment analysis (Figure 1). An alternative approach to explore the Raman spectra shape variations between normal and tumor was to perform signal subtraction of normalized values of mean spectra (10,24). Typical spectral shapes obtained by using this method are presented in Figure 2. Raman spectra obtained from the lung adenocarcinoma and MPNST showed that the most significant differences between normal and tumor tissues in each case appear to be at the 748 cm^-1^ band, which denotes the DNA chain, and in the spectral ranges of 1000-1100, 1200-1400, and 1500-1700 cm^-1^_,_ which contain signals related to the protein and lipid conformations and nucleic acid CH stretching modes. Overlapping of specific vibrational modes of complex biological molecules means that only a few narrow spectral bands can be conclusively assigned (eg, the band at 748 cm^-1^, Figure 2). Assigning the exact wavenumber of the Raman shift for a number of other spectral bands (eg, the band at 1223 cm^-1^, Figure 2A or the band at 909 cm^-1^, Figure 2B) was rather unreliable as these bands were broad and any assignment had the uncertainty up to at least ±2 cm^-1^.

**Figure 3:**
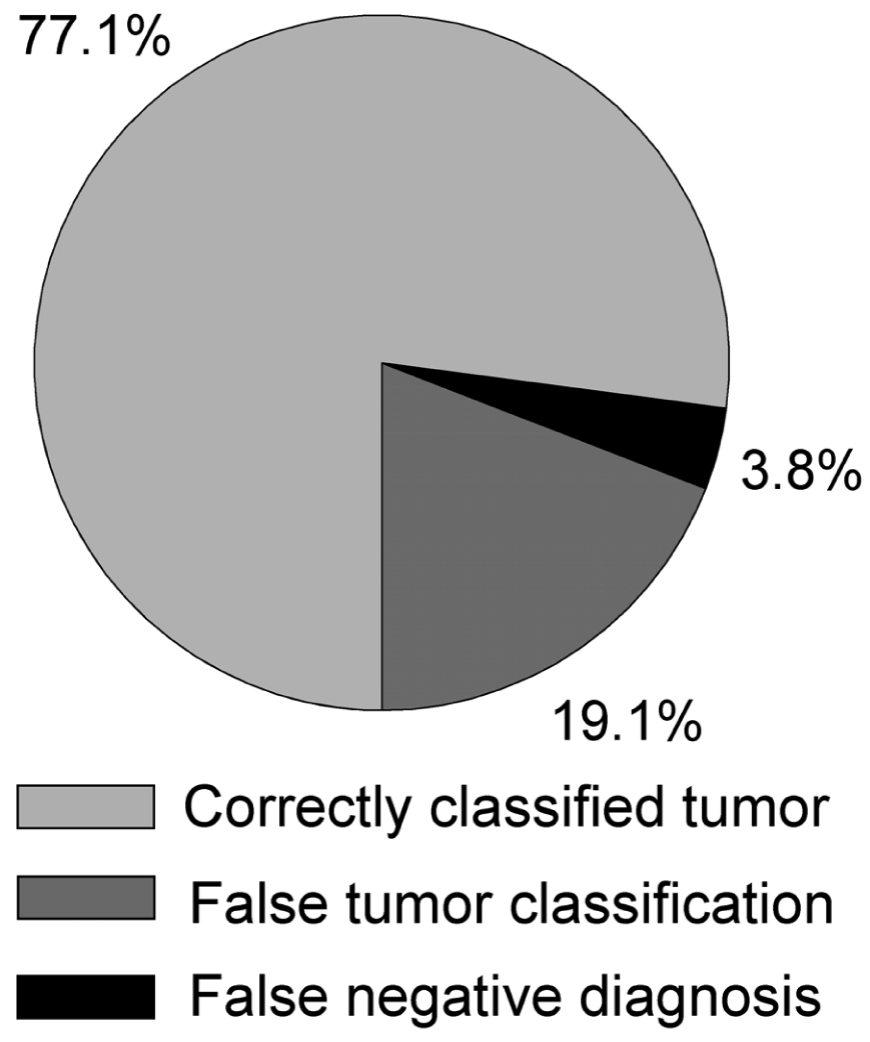
Success score chart of trained neural network for detection of different types of tumors for all recorded spectra.

Results of PCA/NN analysis are presented in Figure 3 and Table 1. While inspecting these results, it has to be remembered that classification was performed on the basis of an individual spectrum from tumors, which was blindly checked against all normal tissues and all other tumors regardless of the specific tissue or tumor. This is a more stringent test for the performance of the PCA/NN analysis than the previously published analysis where the performances have been tested only for a certain tissue and a few selected tumors characteristic for that tissue (16,17).

**Table 1:** Classification success for different types of tumors. Percentage denotes the number of correctly classified tumors, false negative, or false tumor classification

Tumor type (No. of patients)	Classified as:	(%)
Lung squamous carcinoma (3)	Lung squamous carcinoma	83.3
	False negative diagnosis	16.7
Lung adenocarcinoma (3)	Lung adenocarcinoma	91.7
	False tumor classification	8.3
Lung small cell carcinoma (4)	Lung small cell carcinoma	81.3
	False tumor classification	18.8
Chondrosarcoma (3)	Chondrosarcoma	91.7
	False tumor classification	8.3
Osteosarcoma (2)	Osteosarcoma	62.5
	False negative diagnosis	12.5
	False tumor classification	25.0
Ewing's sarcoma (2)	Ewing's sarcoma	75.0
	False tumor classification	25.0
Chondroblastoma (2)	Chondroblastoma	62.5
	False negative diagnosis	12.5
	False tumor classification	25.0
Rhabdomyosarcoma (2)	Rhabdomyosarcoma	75.0
	False tumor classification	25.0
Synovial sarcoma (2)	Synovial sarcoma	62.5
	False tumor classification	37.5
Malignant peripheral nerve sheath tumor (2)	Malignant peripheral nerve sheath tumor	62.5
	False tumor classification	37.5
Hepatocellular carcinoma (2)	Hepatocellular carcinoma	100

Overall sensitivity, defined as differentiation between normal and tumorous tissue (regardless of the type of malignancy), is around 96% (Figure 3.) ie, PCA/NN analysis correctly identified 96% of individual spectra as tumors. Only around 4% of all tumor spectra were incorrectly classified as normal tissue (false negative).

Specificity is usually defined as the ability of the diagnostic procedure to distinguish malignant from benign tumors and/or between different malignant (or benign) tumors. Here, specificity was calculated as a number of spectra that are correctly classified as a particular tumor type vs total number of spectra classified as tumors (96% of all tumor spectra). Our study gave the overall specificity of around 82%. Table 1 summarizes results obtained for individual tumor types.

## Discussion

Obtained results for main and subtracted spectra were mainly in accordance with the previously published data for tumors (1,10,24) using the same procedures. The main problem in differentiating tumors from normal tissue using such data are the presence of numerous broad bands, which makes using the automatic peak assignment procedure unreliable. Some rather subjective procedures have been previously performed to establish the fingerprint wavenumber of these bands (4,7,10,24), but it is obvious that the entire shape of such bands has to be considered when analyzing Raman spectra.

Consequently, full individual Raman spectra were analyzed using combined PCA/NN analysis since it has been demonstrated that NN analysis of Raman spectra showed a superior performance compared with traditional linear models such as multiple linear regressions, spectral library searching, and partial least squares and cluster analysis methods (21,25,26). Successfulness of any method used for tumor diagnosis is characterized by its sensitivity and specificity. Here we achieved sensitivity of 96% in distinguishing tumors from normal tissue and specificity of 80% in distinguishing between various tumor types.

The comparison with other studies might not be straightforward since different authors employed different definitions of sensitivity, nevertheless here are some examples. Skin cancers: 85% (16), 96% (17); bladder cancer: 78.5% (8), and 94% (7); renal tumors 82% (6), lungs 94% (24). Consequently our results are comparable to other studies despite the fact that they were obtained for a wide range of tumors. An exceptionally high sensitivity of 99.5% has been reported for human uterine cervix (9), but this study analyzed cancer at a very advanced stage.

Individually, the sensitivity in the group of lung carcinomas was 95%, where only lung squamous cell carcinoma (SCC) showed false negative classification. This is in accordance with 94% reported in a previous study (24), where just two types of tumors were analyzed (adenocarcinoma and SCC). Again, only SCC showed false negative findings. These findings can be explained by the fact that SCC is formed through squamous metaplasia of normal cylindric epithelium, while other analyzed lung carcinomas have diffusive and infiltrative growth. As a consequence, SCC has a pronounced stromal component so that spectra classified as normal tissue could have been recorded in such areas. Sensitivity for the group of the bone and soft tissue tumors was 96%. We were unable to find any RS study examining these tumors, but high sensitivity obtained here clearly shows the potential of RS in histopathology of these tumors. Sensitivity for both MPNST and hepatocellular carcinomas is 100%, but such a high score may be the consequence of a small number of patients or high-grade malignancy of analyzed tumors.

Reported specificities were 99% for differentiating malignant melanoma (18) or 100% basal cell carcinoma (15) from other benign skin lesions; 87% for different malignant renal tumors (6); 78.9% (8) and 92% (7) for bladder cancers; and 92% for lung cancers (24). Specificity of our study might appear low when compared to other studies, but this is the consequence of our specific approach. For example, some MPNST spectra were incorrectly classified as Ewing’s sarcoma and vice versa. This can be a consequence of the presence of Homer-Wright rosettes, which are usually present in both tissues. Some lung adenocarcinoma spectra were classified as chondrosarcoma, while some lung small cell carcinoma spectra were classified as chondroblastoma, which can be a consequence of the presence of cartilaginous bronchial tissue within the sample. All these problems in differential diagnosis can be easily overcome in a real clinical setting where some misclassifications can be easily eliminated based on the frequency of occurrence of various tumors in a given tissue.

This work, as well as other reports, clearly shows that straightforward inspection of Raman spectra in attempting to differentiate tissues is virtually impossible due to the complexity of tumors and surrounding tissues (16,17). The molecular origin of almost all Raman peaks is reasonably well known; however spectra from tissues are too complex due to the overlap of peaks and simple finger-printing of tissues based on a certain band or a part of spectrum is uncertain. The PCA takes into account all spectral shapes and extracted PCA scores represent the weight of characteristic spectral components of the source spectra. Coupled with the NN classification, such an approach provides an automated diagnostic procedure and is essentially different from the “silver-bullet” approach. Also, diagnosis based on RS is different from standard histopathology since it is done on a molecular level by taking into account virtually all macromolecules within certain tissue.

The use of Raman microscopy has several advantages over the standard RS. First, areas of unwanted, high fluorescence can be easily identified prior to acquiring spectra which significantly reduces the time needed for collecting the amount of spectra necessary for tissue characterization. Second, samples on the millimeter scale are sufficient for RS which enables analysis of samples obtained by fine-needle biopsy.

Our results show that sensitivity and specificity of analysis of spectra obtained from numerous different tumors and tissues is at least comparable to data where only one tissue containing one or a few types of tumors were investigated. The implication of this result is that it seems feasible to start to create an extensive Raman spectra database encompassing a wide variety of tumors and healthy tissues. This can greatly improve differential diagnostic capabilities of Raman spectroscopy based histopathology in clinical settings.
